# To What Extent Do Musculoskeletal Ultrasound Biomarkers Relate to Pain, Flexibility, Strength, and Function in Individuals With Chronic Symptomatic Achilles Tendinopathy?

**DOI:** 10.3389/fresc.2021.726313

**Published:** 2021-08-12

**Authors:** Mathieu Lalumiere, Sarah Perrino, Marie-Josée Nadeau, Christian Larivière, Martin Lamontagne, François Desmeules, Dany H. Gagnon

**Affiliations:** ^1^Faculty of Medicine, Université de Montréal, Montreal, QC, Canada; ^2^Centre for Interdisciplinary Research in Rehabilitation of Greater Montreal (CRIR), Montreal, QC, Canada; ^3^Institut de recherche Robert-Sauvé en santé et en sécurité du travail (IRSST), Montreal, QC, Canada; ^4^Centre de recherche de l'Hôpital Maisonneuve-Rosemont (CRHMR), Montreal, QC, Canada

**Keywords:** computer-assisted image analysis, diagnostic imaging, image processing, musculoskeletal – disorders, rehabilitation, tendinosis, tendinopathy, ultrasonography

## Abstract

**Introduction:** Achilles tendinopathy (AT) is a chronic musculoskeletal pathology best evaluated by ultrasound imaging. This cross-sectional study aimed at better understanding the relationship between musculoskeletal ultrasound biomarkers (MUBs) of Achilles tendon and localized pain, ankle flexibility, ankle strength, and functional abilities.

**Method:** Forty-one participants with unilateral midportion chronic AT had their tendon images analyzed bilaterally in the longitudinal and transverse planes. The Victorian Institute of Sport Assessment-Achilles questionnaire (VISA-A) and Lower Extremity Functional Scale (LEFS) assessed pain and function, respectively, during standing and walking-related activities. Ankle flexibility was evaluated by weight-bearing lunge tests, while ankle isometric peak strength was measured using an instrumented dynamometer. Achilles tendon ultrasonographic images were analyzed using geometric (thickness), composition (echogenicity), and texture (homogeneity) MUBs. Discriminative validity was evaluated using paired Student's *t*-tests to compare MUBs between symptomatic and asymptomatic sides. Predictive validity was evaluated by computing the Pearson product-moment correlations coefficient between MUBs and pain, ankle flexibility, ankle strength, and function.

**Results:** Significant differences were found in MUBs between the symptomatic and asymptomatic sides, confirming the discriminative validity of the selected MUBs. On the symptomatic side, thickness was found 29.9% higher (*p* < 0.001), echogenicity 9.6% lower (*p* < 0.001), and homogeneity 3.8% higher (*p* = 0.001) when compared with the asymptomatic side. However, predictive validity was scarcely confirmed, as most of the correlation coefficients were found negligible for the associations investigated between MUBs with localized pain, ankle flexibility, strength, and function. Only 14 statistically significant low to moderate associations were found, with negative and positive correlations ranging between −0.31 and −0.55 and between 0.34 and 0.54, respectively.

**Discussion:** Musculoskeletal ultrasound biomarkers have a clinical utility in visualizing *in vivo* tendon integrity and diagnosing AT. MUBs should be valued as part of a comprehensive neuro-musculoskeletal assessment as they complement pain, flexibility, strength, and function measures. Altogether, they may inform the development and monitoring of a personalized rehabilitation treatment plan.

## Introduction

Achilles tendinopathy (AT) is a common musculoskeletal pathology that affects the strongest and largest tendon of the human body (1–3). AT generally affects either the midportion of the Achilles tendon or its enthesis on the calcaneus among both sedentary and athletic individuals (1–4). The prevalence of AT increases with age, and AT mostly affects males between 30 and 50 years of age. Although, the precise etiology of AT remains uncertain, the risk of injury is mainly associated with a maladaptive response to overstimulation caused by repetitive plantarflexion and high forces transmitted through the tendon during functional activities involving plantarflexion (5). Other factors such as the presence of common chronic diseases (i.e., diabetes, rheumatoid arthritis, or hypercholesterolaemia), training errors, cold environments, or the use of specific medications (i.e., corticosteroids, fluoroquinolone, statins) may also increase the risk of AT (4, 6, 7). AT-related symptoms typically include pain at rest, pain during or after physical activities, and a feeling of stiffness of the Achilles tendon after a period of immobility. AT main impairments include reduced flexibility of the triceps surae muscles (i.e., gastrocnemius and soleus) and a decrease in both force and endurance of triceps surae muscles (mostly the soleus) (5, 8, 9). These symptoms and impairments typically lead to functional limitations when walking, running, using stairs, and jumping, leading to decreased social participation and quality of life (4, 10, 11).

In chronic AT, episodes of excessive mechanical stress cause pathological changes in the tendon that are visible and distinctive on ultrasound images (4, 12, 13). Affected tendons typically present abnormal collagen and extracellular matrix composition and structure (14). These abnormalities of the Achilles tendon can be quantified by musculoskeletal ultrasound biomarkers (MUBs), such as geometric (thickening), composition (hypoechogenic), or texture (increased homogeneity) dimensions (15–18). MUBs extracted from ultrasound imaging have good reliability when characterizing the biological integrity of the Achilles tendon (19). Furthermore, MUBs allow differentiating between several pathologies (e.g., partial tear, bursitis, peritendinitis) when clinical evaluation is not conclusive (4). Therefore, MUBs are tools frequently used in both clinical and research settings when characterizing the Achilles tendon integrity (20–24).

Interestingly, bodies of evidence are conflicting regarding associations between MUBs and pain, flexibility, strength, and function (25–32). Some authors found a lack of association between anatomopathology and symptom severity in cross-sectional studies (26, 27) or limited structural improvement of the tendon despite clinical improvement in terms of pain and function among individuals with midportion AT during prospective studies (33–35). Inversely, some authors found a significant association between tendon thickness, hypoechogenicity, and pain among cross-sectional studies (25) and prospective studies (36, 37). Consequently, there is no consensus regarding the association between MUBs and clinical outcomes among individuals with AT.

The main objective of the study was to gain knowledge and a better understanding of the relationship between geometric (i.e., thickness), composition (i.e., echogenicity), and texture (i.e., homogeneity) MUBs with localized pain, ankle flexibility, ankle strength, and standing-related functional abilities (e.g., walking, hopping, stairs, sports) among individuals with symptomatic midportion unilate×|<ral AT. More specifically, in terms of discriminative validity, MUBs of symptomatic Achilles tendons were generally expected to appear on images as thicker (i.e., increased thickness), darker (i.e., reduced echogenicity), and smoother (i.e., increased homogeneity) in comparison with the asymptomatic Achilles tendons (15). Moreover, in terms of predictive validity, absolute (i.e., symptomatic tendon) or relative (i.e., symptomatic/non-symptomatic tendon difference) MUBs were expected to be, at least, moderately associated with localized pain, ankle flexibility, strength impairments, and functional disabilities.

## Method

### Study Design

This validity study builds on a previously published one that proposed a minimal data set of MUBs to characterize Achilles tendon health (15). Additional clinical and laboratory measures, which may theoretically relate to MUBs, are now examined to test the hypotheses formulated above.

### Participants

A group of 41 individuals with unilateral midportion chronic AT participated in the study (Table 1). Out of these 41 individuals, additional measures [i.e., Lower Extremity Functional Scale (LEFS) score and peak isometric ankle strength] were collected for the last 20 participants, as the research protocol was updated. Hence, these last 20 participants form a distinct subset of participants (i.e., subgroup). To be included in the study, potential participants had to report unilateral and localized pain in the Achilles tendon for more than 3 months (6), experience pain on palpation of the midportion of the Achilles tendon, and achieve a score below 90 out of 100 on the Victorian Institute of Sport Assessment-Achilles Questionnaire (VISA-A) (38). Potential participants who reported bilateral pain during activities had a body mass index (BMI) >30 kg/m^2^ and reported a history of Achilles tendon rupture, were diagnosed with a metabolic, neurologic, or systemic inflammatory disease, or had received any type of injection in the Achilles tendon in the past year were excluded.

**Table 1:** Mean (SD) characteristics of the participants.

	**Units**	**Group**	**Subgroup**	***p*** -value^*^
		***n*** **= 41**	***n*** **= 20**	
**Sociodemographic and anthropometric measures**				
Age	years	42.5 (8.6)	42.2 (8.4)	0.911
Sex - male/female	*n*	27/14	13/7	0.949
Height	cm	1.74 (0.08)	1.75 (0.09)	0.880
Weight	kg	78.2 (15.4)	76.6 (15.3)	0.692
BMI	kg/m^2^	25.7 (4.7)	25.0 (4.6) 0.602	
Symptomatic side - left/right	*n*	24/17	12/8	0.915
Time since injury	months	26.4 (29.9)	24.0 (18.7)	0.740
Time since injury - range	Min–Max	3–120	3–85	N/A
**Pain and functional measures**				
VISA-A score	/100	60.7 (18.6)	59.5 (19.0)	0.810
VISA-A-range	Min–Max	13–82	13–82	N/A
*Q1 stiffness*	/10	7.0 (3.2)	6.8 (2.9)	0.791
*Q2 stretching*	/10	6.6 (3.2)	6.0 (3.2)	0.473
*Q3 pain walking*	/10	7.4 (2.6)	7.1 (2.7)	0.642
*Q4 pain stairs*	/10	7.5 (2.7)	7.6 (2.4)	0.903
*Q5 pain heel raise*	/10	6.3 (2.9)	6.6 (2.6)	0.740
*Q6 pain hopping*	/10	4.4 (3.4)	4.5 (3.8)	0.909
*Q7 sports level*	/10	5.8 (2.5)	5.6 (2.7)	0.744
*Q8 sport time*	/30	15.6 (7.8)	19.3 (11.4)	0.147
LEFS	/80	N/E	63.9 (10.6)	N/A
LEFS - range	Min–max	N/E	38–78	N/A

*BMI, body mass index; VISA-A, Victorian Institute of Sport Assessment-Achilles Questionnaire; LEFS, lower extremity functional Scale; N/E, not evaluated; N/A, not applicable*.

* *Usually by independent bilateral Student's t-tests; otherwise for sex and symptomatic side by chi-squared test. Statistical significance set at a level of p < 0.05*.

### Clinical Evaluations

Clinical evaluations were conducted by two trained experienced physiotherapists (ML and MJN) using a standardized data collection protocol.

#### Sociodemographic, Anthropometric, Pain, and Functional Assessments

Basic sociodemographic (i.e., age, sex) and anthropometric data (i.e., height, weight) were collected first before documenting AT-related information (e.g., the side affected, time since the first symptom, etc.,). The participants then completed one or two patient-reported outcome measures depending on when they entered the study. All the participants filled out the VISA-A (39). This questionnaire includes eight questions targeting three dimensions: localized pain in the Achilles tendon, function in daily life, and participation in sports activities (38–40). Questionnaire scores range from 0 to 100, with a low score indicating greater severity of the impact of the AT. The questionnaire is available in English and French, and it is reliable, valid, and sensitive to change with a minimal clinically important difference (MCID) of between 6 and 20 points (38, 41). The LEFS (42) questionnaire was only completed by the participants in the subgroup. The LEFS assesses the function of individuals with orthopedic disorders affecting their lower extremities, such as AT (42). The LEFS includes 20 questions measuring the level of difficulty encountered when performing activities of daily living and sports. The LEFS has a maximum score of 80, with a higher score confirming a higher functional level (42). The LEFS, which is also available in English and French, is reliable, valid, and sensitive to changes with a MCID of 9–12 points (41). The VISA-A and LEFS were completed either on paper or electronically *via* the Lime Survey® platform in the choice of language (French or English) of the participants.

#### Ankle Flexibility

Mono- and bi-articular muscle flexibility tests of the ankle were performed by the *front-limb weight-bearing lunge test* to assess the soleus and the *back-limb weight-bearing lunge test* with the knee extended to assess the flexibility of the gastrocnemius muscles (43, 44). For these two tests, the distance between the tip of the hallux and the wall was measured in cm. A reduction in flexibility of the soleus would translate to a decrease in the distance between the wall and the hallux of the front limb, whereas, reduced flexibility of the gastrocnemius muscles would translate to a decrease in the distance between the wall and the hallux of the back limb.

#### Isometric Strength

Peak isometric ankle strength, assessed exclusively in the subgroup, was measured using a Biodex instrumented dynamometer (Biodex Medical Systems, Shirley, NY, United States). The participants sat in a multi-position adjustable chair, with their hip flexed to 135°, knee extended, and the ankle firmly attached to a custom-made boot attached to the dynamometer to isolate ankle movement and prevent the heel from lifting during plantarflexion (45). The ankle was positioned at 10° plantarflexion during the isometric strength assessment (46). The physiotherapist (ML), assisted by a physiotherapy student (SP), instructed the participants to gradually contract their plantarflexor muscles up to their peak strength then hold the contraction for about 2 s (a total of ~5 s of contraction), while standard verbal encouragement was provided to ensure maximal effort throughout the tests (47, 48). Two trials were recorded, and a third one was recorded whenever the difference was >10% between the first two trials. A 1-min break was allowed between each trial. Each participant familiarized themselves with the dynamometer prior to the assessment (47, 49). The same process was applied for measuring isometric dorsiflexion force, but with the foot at 20° plantarflexion. The mean peak moment was computed for plantarflexion and dorsiflexion separately for both the symptomatic and asymptomatic sides. A dynamometer provided reliable and valid measures to characterize the strength-generating capability at the ankle (50, 51), and to differentiate individuals with good tendon health from those with AT (21, 52).

#### Musculoskeletal Ultrasound Imaging

##### Image-Acquisition

**Figure 1:**
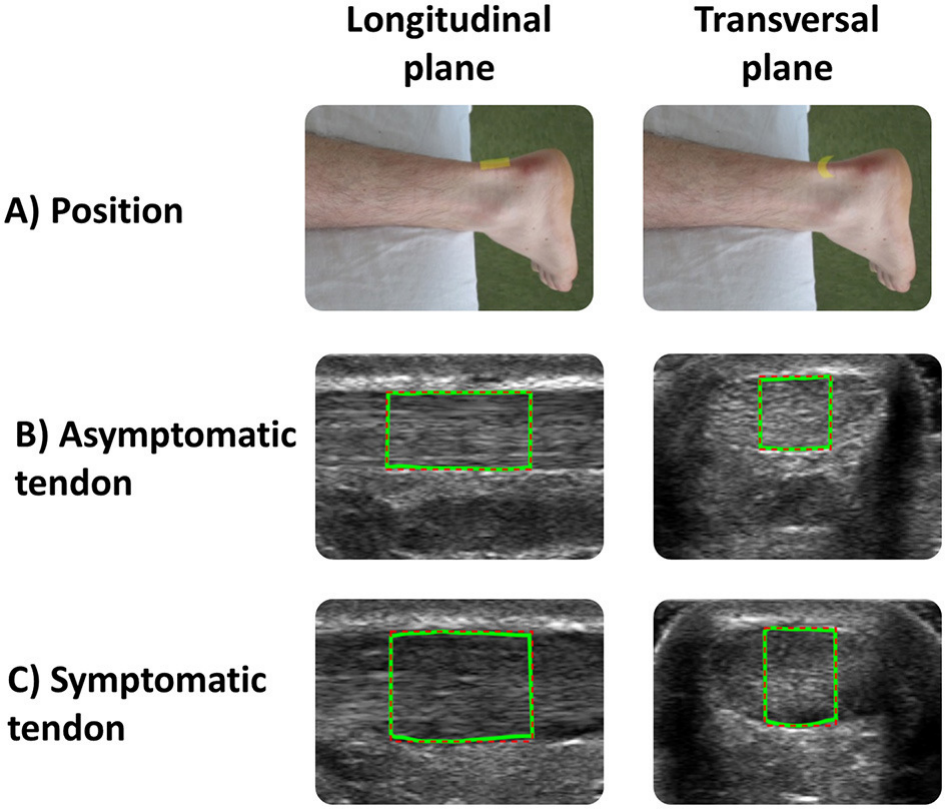
**(A)** Positions of the transducer in the longitudinal and transverse planes used to acquire asymptomatic and symptomatic Achilles tendon ultrasound images. The green lines correspond to the region of interest (ROI) used for data analysis for **(B)** asymptomatic and **(C)** symptomatic tendon.

All the ultrasound images of the Achilles tendon were acquired in brightness mode using a 5–12 MHz linear array transducer with a 5-cm wide footprint connected to an HD 11XE 1.0.6 ultrasonography system (Phillips Medical Systems, Bothell, WA, United States). A previously described standardized protocol (15, 19) was used for image acquisition. All parameters affecting image quality (i.e., probe frequency set at 12 MHz; depth = 2 cm; gain = 85; unique focus zone adjusted to 0.5 cm at the AT level; neutral time gain compensation) remained constant during each data collection session and across the participants. In addition, care was taken to align the transducer parallel to fiber orientation to minimize anisotropy. The most painful region along the posterior aspect of the symptomatic tendon was first located by palpation before its location was marked on the skin and mirrored on the asymptomatic side. The probe was then positioned at the marked site to view the Achilles tendon fibers in the longitudinal and transverse planes, respectively (Figure 1A). Three images were acquired in both the longitudinal and transverse planes (*n* = 6 images per side). The probe was removed after the recording of each image and always repositioned at the marked site thereafter.

##### Image-Analysis

**Figure 2:**
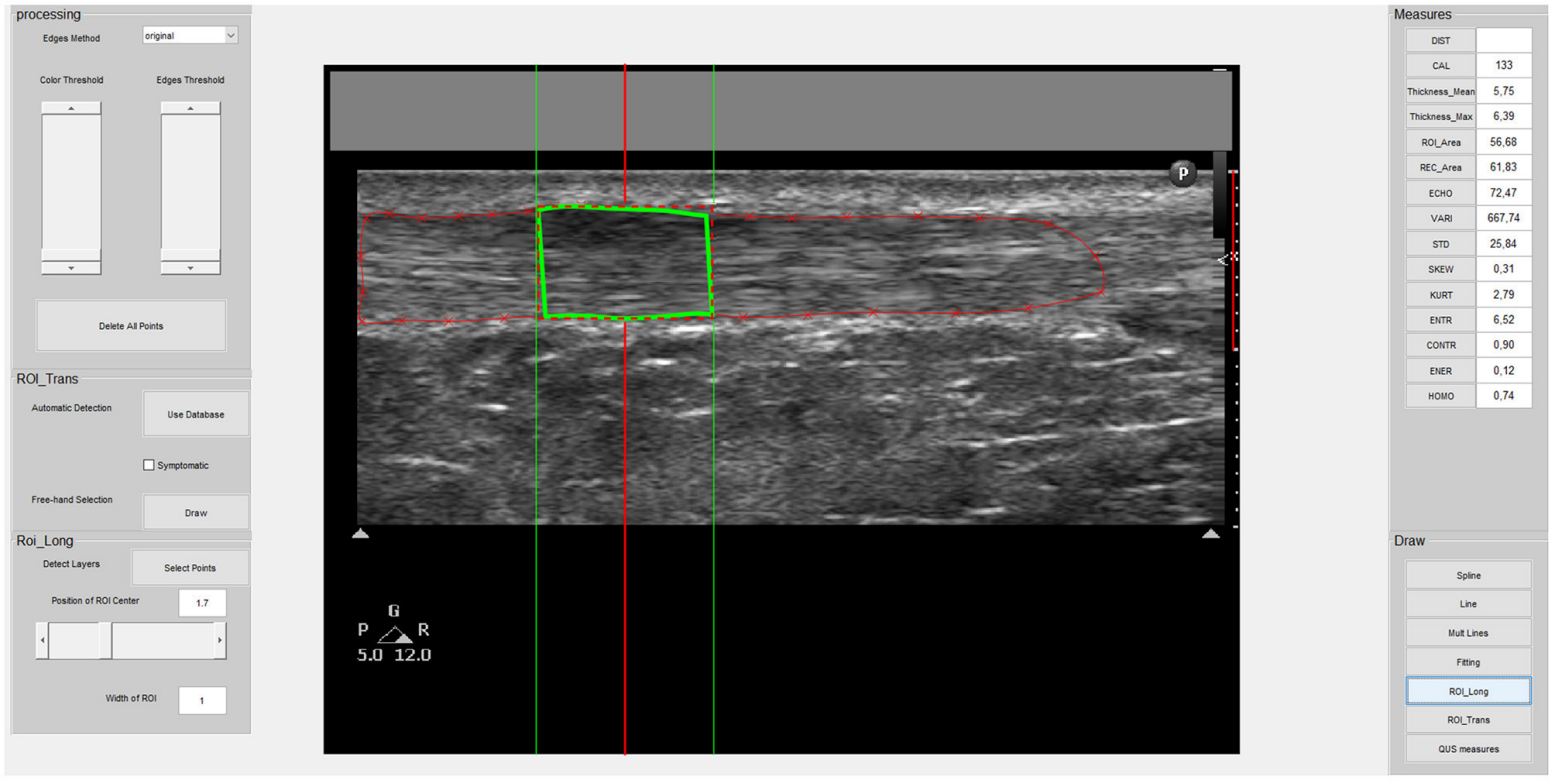
Screenshot of the custom-made program interface used to select the ROI and acquire musculoskeletal ultrasound biomarker (MUB) measures of the Achilles tendon image. This is an example of a symptomatic tendon in the longitudinal plane with a 1-cm wide ROI.

All the images were analyzed using a custom interactive 2D viewing and image analysis program developed by the research team with Image Processing Toolbox™ from MATLAB (MathWorks Inc., Natick, MA, United States), as used in previous studies (19, 53). Using this program, a region of interest (ROI) was manually outlined within the hyperechoic margins of the tendon (i.e., circumferential epitenon) (Figure 2). A semi-automatic tracing procedure then selected the lateral margins of the 1-cm and 0.5-cm wide ROIs in the longitudinal and transverse planes, respectively (Figures 1B,C). In the transverse plane, this process also reduces lateral anisotropy and consequently improves the reliability of MUBs (53, 54). Based on the previously recommended minimal dataset organized around three dimensions (i.e., geometric, composition, and texture), MUBs were computed for each ROI taken in the longitudinal and transverse planes (i.e., mean thickness, echogenicity, and homogeneity at 90° in the longitudinal plane and multidirectional mean in the transverse plane). MUB values of the three measurements were averaged for each plane. Additional details on the various measures are available elsewhere (19).

### Statistical Analyses

Descriptive statistics (e.g., mean, SD, proportion, range) synthesized the demographics, and the anthropometric clinical and laboratory outcomes. The normality of the distribution of outcomes was verified by the Shapiro–Wilk test and using a graphical method. Differences between the main group (*n* = 41) and the subgroup (*n* = 20) demographic, pain, and functional measures were compared by bivariate independent Student's *t*-test for parametric data and the chi-squared test for non-parametric data. Differences between the asymptomatic and symptomatic sides for clinical (i.e., flexibility and strength) and ultrasound (i.e., mean thickness, echogenicity, and homogeneity) measures were assessed by independent paired Student's *t*-tests and Wilcoxon signed-rank tests for normally and non-normally distributed data, respectively. Effect sizes were also computed using Hedge's g (55) to determine the absolute magnitude of the effect sizes: >0.2 was considered small, >0.5 was considered medium, and >0.8 was considered large (56). Thereafter, a difference index (DI) was computed to assess the relative difference between the symptomatic and asymptomatic sides for all clinical and ultrasound measures using Equation 1:


(1) $$\begin{array}{r} Difference\;Index\;\left(\%\right)=\frac{\left(Symptomatic-Asymptomatic\right)}{\left(Asymptomatic\right)}\;\times \;100 \end{array}$$

Last, the association between the MUBs and the absolute (i.e., symptomatic side) and relative (i.e., DI) measures of localized pain (i.e., VISA-A), ankle flexibility and strength impairments, and functional disabilities (i.e., VISA-A and LEFS) were assessed using Pearson product-moment correlation and Spearman rank-order correlation for normally and non-normally distributed data, respectively. The strength of correlation coefficients was considered negligible between 0 and .3, low between 0.3 and 0.5, moderate between 0.5 and 0.7, and high between 0.7 and 0.9 (57). The threshold for statistical significance was set at 0.05 for all statistical analyses carried out with the SPSS v25 software.

## Results

### Characteristics of Participants

A summary of the characteristics of the participants and scores achieved on the VISA-A and LEFS questionnaires are presented in Table 1. Participant characteristics were not significantly different (*p* = 0.147 to 0.949) between the main group and the subgroup.

### Clinical Measures

A summary of the functional measures is presented in Table 1, whereas, the clinical measures and MUBs are summarized in Table 2. Only flexibility measures for the soleus revealed a significant (*p* = 0.002) but the small between-side difference (Hedge's g of −0.23) in soleus flexibility. The soleus on the symptomatic side was 7.3% less flexible than the one on the asymptomatic side. The flexibility of the gastrocnemius (*p* = 0.245) and the isometric strength of the ankle plantarflexors (*p* = 0.182) and dorsiflexors (*p* = 0.52) were similar between the asymptomatic and symptomatic sides.

**Table 2:** Mean (SD) clinical measures, musculoskeletal ultrasound biomarkers (MUBs), and difference index (DI) between sides.

	**Asymptomatic**	**Symptomatic**	**DI (%)**	**Effect size (g)**	***p*** -value^*^
**Clinical measures**					
Flexibility					
Soleus (cm)	11.6 (3.5)	10.8 (3.8)	−7.3	−0.23	0.002^†^
Gastrocnemius (cm)	70.6 (11.3)	68.5 (10.9)	−2.9	−0.18	0.245
Isometric strength – subgroup					
Plantarflexion (N·m)	139.0 (30.3)	133.9 (35.2)	−3.7	−0.15	0.182
Dorsiflexion (N·m)	43.6 (10.6)	42.5 (10.5)	−2.5	−0.10	0.520
**Musculoskeletal ultrasound biomarkers**					
Longitudinal plane					
Mean thickness (mm)	5.15 (1.30)	6.69 (1.75)	29.9	0.99	<0.001^*^
Echogenicity (/255)	77.0 (15.7)	69.6 (13.3)	−9.6	−0.51	<0.001^*^
Homogeneity at 90°	0.595 (0.023)	0.618 (0.029)	3.8	0.86	0.001^*^
Transverse plane					
Mean thickness (mm)	5.17 (1.13)	6.48 (1.78)	25.3	0.87	<0.001^*^
Echogenicity (/255)	83.7 (15.4)	78.3 (14.6)	−6.4	−0.35	0.005^*^
Mean homogeneity	0.621 (0.025)	0.644 (0.032)	3.7	0.78	<0.001^*^

* *Usually by paired Student's t-tests; otherwise (†) for soleus flexibility by Wilcoxon signed ranks tests. Statistical significance set at a level of p < 0.05*.

### Musculoskeletal Ultrasound Biomarkers

A summary of the MUBs is presented in Table 2. The mean thickness of the Achilles tendon revealed a significant (*p* < 0.001) and large between-side difference (g = 0.99 and 0.87), reaching +29.9 and +25.3% for the symptomatic tendon when compared with the asymptomatic tendon in the longitudinal and transverse planes, respectively. The echogenicity of the tendon revealed a significant (*p* < 0.001 and *p* = 0.005) and medium to small between-side difference (g = −0.51 and −0.35), reaching −9.6 and −6.4% for the symptomatic tendon when compared with the asymptomatic tendon in the longitudinal and transverse planes, respectively. The homogeneity of the tendon revealed a significant (*p* < 0.001 and *p* < 0.001) and large to medium between-side difference (g = 0.86 and 0.78), reaching 3.8 and 3.7% for the symptomatic tendon when compared with the asymptomatic tendon in the longitudinal and transverse planes, respectively.

### Associations Between MUBs and Other Measures

**Figure 3:**
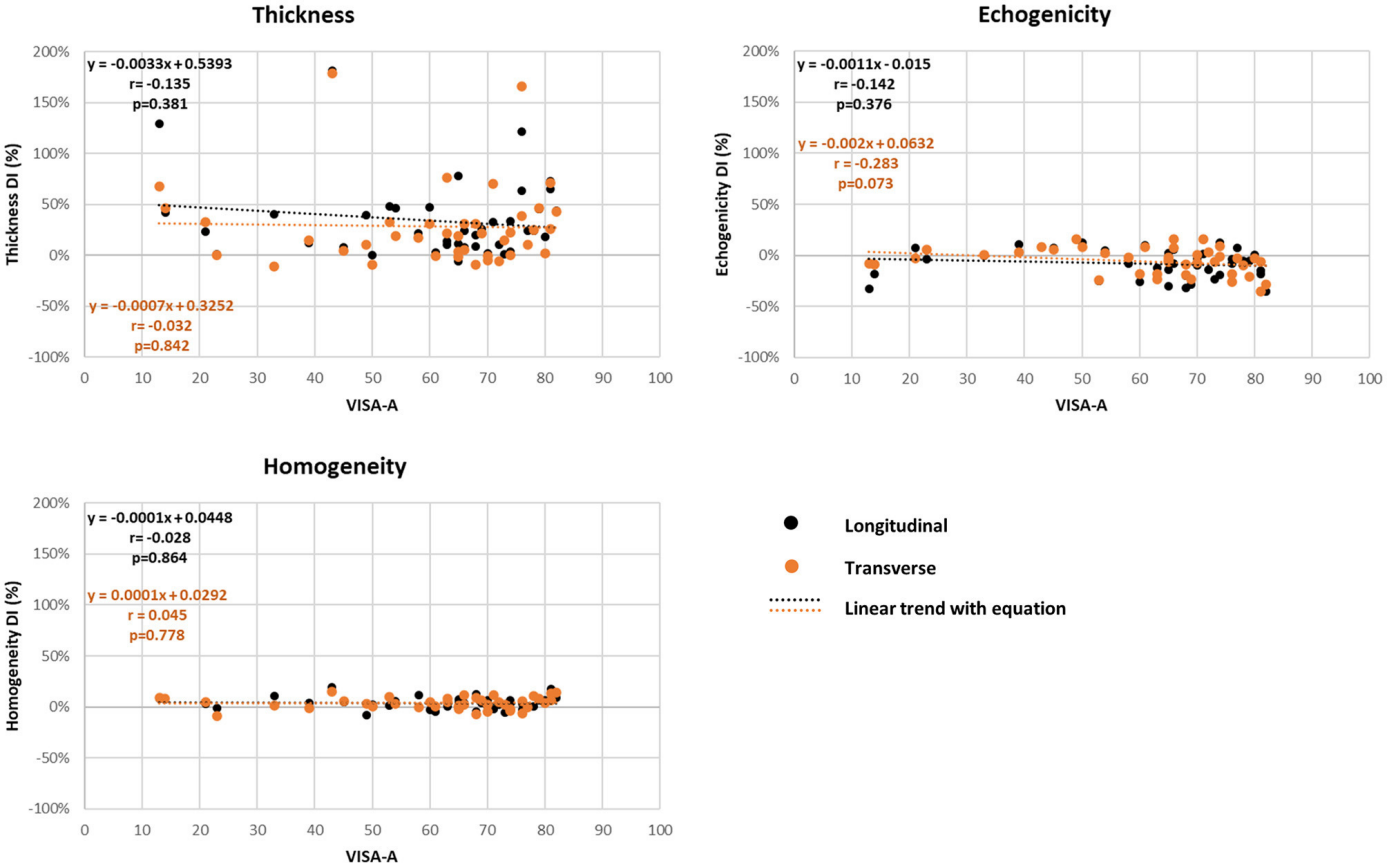
Correlation (r) of ultrasound biomarker difference index (DI) measures and Victorian Institute of Sport Assessment-Achilles Questionnaire (VISA-A) total score (Pearson product-moment test).

A summary of the associations between the MUBs and clinical and functional measures is presented in Table 3 and Figure 3. Overall, the correlation coefficients were found to be negligible for most of the associations investigated. Only few statistically significant low to moderate correlations were found (*n* = 14, negative and positive correlations ranging between −0.31 and −0.55 and between 0.34 and 0.54, respectively). Among the clinical measures, low correlations were found between the flexibility of the gastrocnemius in both planes and the absolute thickness-Sympt and echogenicity-Sympt. Also, low to moderate correlations were found between the ankle plantarflexor strength-DI and the echogenicity-DI in the longitudinal plane and between the ankle dorsiflexor strength-Sympt and the mean thickness in both planes. Among the pain and functional measures, the mean thickness of the symptomatic tendons in both planes was consistently correlated with questions #7 and #8 of the VISA-A, suggesting higher limitations in sports level and time as tendon thickness increases. For these two questions, albeit not as consistently correlated as for thickness, few echogenicity and homogeneity measures were also found to be significantly associated. In the longitudinal plane, homogeneity and question #5 – *painful heel raise* as well as between echogenicity and question #6 – *pain during hopping*. No significant correlations (i.e., low, moderate, or high) were observed between the total VISA-A score and MUBs (Figure 3), or between the LEFS questionnaire and the MUBs.

**Table 3:** Associations between MUBs and clinical measures for the symptomatic (Sympt) side and DI separately.

	**Longitudinal plane**						**Transverse plane**					
	**Mean thickness**		**Echogenicity**		**Homogeneity at 90** ^**°**^		**Mean thickness**		**Echogenicity**		**Mean homogeneity**	
	**Sympt**	**DI**	**Sympt**	**DI**	**Sympt**	**DI**	**Sympt**	**DI**	**Sympt**	**DI**	**Sympt**	**DI**
**Clinical measures**												
Flexibility												
Soleus - Sympt	0.18		−0.23		0.10		0.17		−0.20		0.12	
Soleus - DI		0.21		−0.01		0.26		0.24		−0.09		0.23
Gastroc - Sympt	0.13		–0.32^*^		−0.07		0.15		–0.32^*^		0.08	
Gastroc - DI		0.06		−0.11		0.00		0.05		−0.27		−0.06
Isometric strength - Subgroup												
Plantarflexion - Sympt	0.24		−0.06		−0.14		0.12		−0.21		−0.18	
Plantarflexion - DI		−0.17		0.54^*^		−0.26		0.02		0.23		−0.18
Dorsiflexion - Sympt	0.34		−0.02		0.13		0.25		0.00		0.10	
Dorsiflexion - DI		–0.49^*^		0.41		0.03		–0.55^*^		0.26		−0.20
**Pain and functional measures**												
VISA-A score	−0.11	−0.16	−0.10	−0.14	0.03	−0.03	−0.11	−0.03	−0.15	−0.28	0.01	0.05
*Q1 stiffness*	−0.15	−0.07	0.27	0.04	0.05	0.02	−0.07	0.04	0.22	0.15	−0.09	−0.02
*Q2 stretching*	0.01	−0.23	−0.06	−0.07	0.15	−0.06	0.01	−0.20	−0.06	−0.10	0.05	0.04
*Q3 pain walking*	0.15	0.03	0.00	−0.17	0.24	0.07	0.13	0.17	−0.07	−0.22	0.16	0.27
*Q4 pain stairs*	0.20	0.10	−0.22	−0.22	0.23	0.11	0.17	0.16	−0.21	−0.13	0.31	0.19
*Q5 pain heel raise*	0.29	0.00	−0.23	−0.19	0.34^*^	0.16	0.25	0.14	−0.20	−0.31	0.21	0.01
*Q6 pain hopping*	0.19	−0.03	–0.36^*^	0.03	0.20	0.11	0.16	−0.02	−0.29	−0.15	0.17	−0.09
*Q7 sport level*	–0.36^*^	−0.20	0.03	−0.14	−0.22	−0.04	–0.35^*^	−0.18	0.06	−0.22	–0.33^*^	−0.13
*Q8 sport time*	–0.40^*^	−0.22	−0.02	−0.08	–0.31^*^	−0.20	–0.39^*^	−0.11	−0.13	–0.33^*^	−0.16	0.02
LEFS Score	0.01	−0.29	−0.31	0.04	0.02	−0.26	0.23	0.23	−0.24	−0.04	0.22	0.14

* *Usually by Pearson product-moment correlations; otherwise for soleus flexibility by Spearman rank-order correlations. Statistical significance set at a level of p < 0.05*.

## Discussion

In line with previous research studies, this one further explored the discriminative ability of MUBs and their predictive validity with regard to localized tendon pain, ankle flexibility, ankle strength, and standing-related functional abilities among individuals with unilateral symptomatic AT. The results confirm that the discriminative validity of MUBs is high between the symptomatic and asymptomatic sides, whereas, the predictive validity of MUBs with patient-reported outcome measures and clinically related outcome measures are limited and inconsistent across the assessed clinical outcomes (i.e., pain, flexibility, strength, and functional abilities).

### Discriminative Validity

In agreement with the first hypothesis, significant differences were found in MUBs between the symptomatic and asymptomatic Achilles tendons among individuals with chronic symptomatic unilateral AT, confirming the capacity of the selected MUBs to discriminate a symptomatic tendon from an asymptomatic one. Geometric, composition, and texture MUBs change (mostly at the cellular and structural level) in response to localized tendon maladaptive changes as tendinopathy develops (58). In the symptomatic tendon, abnormal tenocyte morphology and changes in proteoglycan content typically increase bound water and tendon thickness in comparison with the asymptomatic tendon (59). In addition, hydrated components of the extracellular matrix increase, and fibrillar disorganization progressively develops, resulting in a reduced quantity of intact collagen fibers and, consequently, reduction in reflected ultrasounds, and production of a darker hypo-echogenic image (13). Finally, fibrillar disorganization triggers changes from type I collagen in favor of type II and III collagens and alters parallel fiber alignment, resulting in alteration of the typical white and gray striated tendon pattern and, thus, smoother texture (i.e., increased homogeneity) (60). Hence, as anticipated, based on previous studies (61–63), the MUBs of symptomatic Achilles tendons generally stood out on the images as being thicker, darker, and smoother in comparison with the asymptomatic Achilles tendons, showing high discriminative validity. These findings further support the conclusions of Matthews et al. (62) who mentioned that geometric and composition MUBs (i.e., thickness and echogenicity) represent key MUBs for assessing images of symptomatic Achilles tendons in clinical and research settings. These findings also reinforce the clinical utility of quantifying echogenicity, instead of only subjectively identifying the presence or absence of hypoechogenic areas (62, 64).

The findings of this study also address a concern raised by Matthews et al. (62) who confirmed the relevance of texture-related MUBs in characterizing tendon matrix changes, although, they are typically lacking in the literature. In that sense, the findings of this study confirm that homogeneity is a MUB that allows clinicians to quantify the fibrillary disorganization of collagen fibers (54) and, as such, may deserve additional attention in future studies. Conversely, the findings of this study provide indirect evidence for the use of a contrast measure as a MUB, since it stands as a relative mathematical inverse of homogeneity in second-order statistics (19). Finally, to build on this study confirming that MUBs are valuable in discriminating a symptomatic tendon from an asymptomatic one (i.e., first iteration), future studies could focus on the classification of symptomatic tendons into tendinopathy stages (i.e., second iteration) as previously attempted (20). In line with this idea, using emerging machine learning and deep-learning approaches may further facilitate collaborative image-based decision-making (65). Achieving such a milestone could add value to the use of MUBs to support the clinical decision-making process.

### Predictive Validity

Contrary to the second hypothesis, the findings of this study highlight the fact that both absolute and relative MUBs have only negligible to low associations with localized tendon pain, ankle flexibility and strength, and functional abilities among individuals with chronic unilateral AT. In fact, it was anticipated that the increased thickness, reduced echogenicity, and increased homogeneity observed at the symptomatic tendon could lead to a better understanding of the vicious cycle in which localized tendon pain, reduced ankle flexibility and strength, and reduced functional abilities and sport performances interact (66). Hence, substantial uncertainty still exists with regard to the predictive validity of MUBs (29, 64), and their added value to support the development of a personalized rehabilitation intervention treatment plan remains controversial (67). Moreover, controversies still exist as to whether or not MUBs can change concurrently to favorable treatment outcomes, as well as how and to what extent they may do so (26, 34, 35, 68).

Regarding pain-related measures, further reflection is needed to explain why they are not, at least, moderately associated with current MUBs in individuals who have chronic unilateral AT. Part of the answer may relate to the increase in angiogenic growth factors, resulting in considerable neovascularization, particularly on the ventral side of the tendon, along with nerve ingrowths that have been proposed as the origin of tendinopathy-related nociceptive pain (69). These peripheral adaptations may increase sensitivity to chemical pain mediators and trigger an over-activation of nociceptors (70, 71). Hence, the inclusion of quantitative Doppler-related MUBs may become indispensable to best capture such adaptions in the future, while also reinforcing their relevance in composite ultrasonography-based scoring when coupled with geometric and composition measures (i.e., thickness and echogenicity) (20, 72). Part of the answer may also relate to sensitization of the central nervous system (i.e., central adaptations), commonly associated with chronic musculoskeletal impairments (73, 74), or psychological factors affecting pain (e.g., pain catastrophizing and kinesiophobia) (75–77), which can both evolve independently to peripheral tendon alterations characterized with MUBs. Another part of the answer may ultimately relate to coexisting plantaris tendinopathy and surrounding tissue alterations, as these conditions affect about one in every 10 individuals with confirmed midportion AT (78, 79).

As for flexibility, strength, and function-related measures, despite tendon changes and the presence of pain expected to alter the loading capacity of the Achilles tendon, only few low to moderate associations were found between these constructs. First, the flexibility of the gastrocnemius on the symptomatic side pointed out a low association between the changes in flexibility and extracellular matrix content (i.e., reduced echogenicity) in both planes. Those results highlight that tendinopathy-related alterations in the Achilles tendon, characterized *via* composition and texture MUBs, do not predict flexibility of the triceps surae muscles (i.e., gastrocnemius and soleus). Second, despite a potentially altered loading capacity highlighted with the MUBs, only the echogenicity-DI was associated with the isometric strength-generating capability of the plantarflexors-DI. Such lack of strong and numerous associations may be explained in part by the fact that no significant difference was revealed between the symptomatic and asymptomatic sides when generating plantarflexion strength. Moreover, it is also plausible that the load generated was insufficient to observe the anticipated difference and, in the future, may warrant greater loading (e.g., eccentric tests, heel raises, or hopping) to gain a better understanding of the association between MUBs and strength (4, 43, 80). In that sense, the *Q5 pain heel raise* on the VISA-A, which coupled heavy load concentric-eccentric contractions, was associated, to some extent, with MUBs. Similar results were observed in a recent cross-sectional study investigating runners with midportion AT, where no significant difference in strength and endurance was found between the symptomatic and asymptomatic sides, although, the symptomatic side showed a significant decrease in plantarflexor strength when compared with healthy matched controls (9). Finally, for the functional outcome measures, the *Q6 sport level and Q7 sport time* on the VISA-A were predicted, to some extent, by some absolute and relative MUBs. This remains plausible considering sport level and maximal sport time may be altered by the loading capacity of the tendon, which changes as the tendon is altered by tendinopathy. Altogether, these results point toward potential central neuroplastic adaptations changing sensory and motor representations in individuals with a chronic musculoskeletal condition, such as in the population of individuals with chronic AT recruited in this study, resulting in bilateral perceptual changes of body image and motor control (73, 81). Ultimately, these adaptations may even trigger structural and histologic changes in the asymptomatic contralateral tendon in individuals with unilateral tendinopathy, providing further evidence for the bilateral nature of tendinopathy (30, 82, 83).

On the whole, the complex interactions between tendon changes and localized tendon pain, ankle flexibility, and strength, and functional abilities among individuals with chronic unilateral AT most likely explain the associations found in this study and definitively warrant further examination (58). Meanwhile, MUBs should be used in conjunction with clinical findings to adjust treatment protocol and improve clinical outcomes (75). Hence, gathering additional information through comprehensive anamnesis and neuro-musculoskeletal assessment continues to be vital for informing rehabilitation professionals in clinical practice and research environments.

### Limitations

Some limitations of this study warrant discussion. First, the relatively modest sample of participants (*n* = 41) with AT, which was further reduced by the fact that ankle strength and LEFS data were only available for about half of the sample (*n* = 20), may have reduced the statistical power, although, moderate-to-large effect sizes were found for most MUBs discriminating the symptomatic and asymptomatic tendons (first hypothesis). Moreover, such sample size may have influenced data distribution and distorted correlation coefficients (second hypothesis). Second, the relatively homogeneous sample of participants with chronic midportion tendinopathy added to the fact that no specific consideration into the possible co-existence of plantaris tendinopathy (e.g., differential diagnosis) was incorporated in the research protocol (84), calls for cautiousness concerning the generalization of the results of this study in the context of an acute or insertional AT. Further caution is also advised if attempting to generalize these findings to other tendons (e.g., patellar tendon, supraspinatus tendon). Third, the fact that the asymptomatic tendon (i.e., contralateral tendon) was used as the denominator (i.e., comparator) to compute the asymmetry index may have attenuated the magnitude of asymmetry, as both tendons may be structurally compromised in individuals with unilateral AT (30). Hence, the inclusion of healthy matched controls could have enriched the findings (81). Finally, the addition of MUBs expressing neovascularization (i.e., Doppler) or stiffness (i.e., elastography) of the tendon could have strengthened the results of this study (85).

## Conclusion

Musculoskeletal ultrasound biomarkers have a clinical utility in visualizing *in vivo* tendon integrity. They add valuable diagnostic information when midportion AT is suspected in clinical practice and research protocols. However, MUBs among individuals with chronic AT are at best weakly associated with pain, flexibility, strength, and functional capacity. In that sense, MUBs are to be valued as part of a multimodal assessment, as they complement patient-reported outcome measures targeting pain and activity limitations and clinically based measures such as ankle flexibility and strength. These last two types of measures may provide greater insight into the symptomatology and functional impacts of chronic AT than MUBs alone and best inform the rehabilitation treatment plan.

## Data Availability Statement

The raw data supporting the conclusions of this article will be made available by the authors, without undue reservation.

## Ethics Statement

The studies involving human participants were reviewed and approved by Centre for Interdisciplinary Research in Rehabilitation of Greater Montreal (CRIR) Research Ethics Committee. The patients/participants provided their written informed consent to participate in this study.

## Author Contributions

MLal and DG contributed to the design of the project, development of the methodology, research ethics approval process, collection of data, validation, analysis and interpretation, writing of the original draft of the manuscript, final review and editing of the manuscript, project administration, and acquisition of funding. SP and M-JN contributed to the acquisition of data, validation and analysis, the writing of the original draft of the manuscript, and the final review and editing of the manuscript. CL, MLam, and FD contributed to the design of the project, development of the methodology, data analysis and interpretation, the final review and editing of the manuscript, and acquisition of funding. All authors contributed to the article and approved the submitted version.

## Conflict of Interest

The authors declare that the research was conducted in the absence of any commercial or financial relationships that could be construed as a potential conflict of interest.

## Publisher's Note

All claims expressed in this article are solely those of the authors and do not necessarily represent those of their affiliated organizations, or those of the publisher, the editors and the reviewers. Any product that may be evaluated in this article, or claim that may be made by its manufacturer, is not guaranteed or endorsed by the publisher.
